# Health Side Story: Scoping Review of Literature on Narrative Therapy for ADHD

**DOI:** 10.3390/healthcare13111247

**Published:** 2025-05-26

**Authors:** Yaakov Ophir, Hananel Rosenberg, Refael Tikochinski, Yaniv Efrati

**Affiliations:** 1 Department of Education, Ariel University, Ariel 4070000, Israel; 2 Centre for Human-Inspired Artificial Intelligence (CHIA), University of Cambridge, Cambridge CB2 1PZ, UK; 3 The Moscowitz School of Communication, Ariel University, Ariel 4070000, Israel; hananelro@gmail.com; 4 Experimental Psychology Department, University College London, London WC1E 6BT, UK; 5 Faculty of Education, Bar-Ilan University, Ramat Gan 5290002, Israel

**Keywords:** ADHD, attention deficit, narrative therapy, child psychotherapy, literature review

## Abstract

Narrative therapy generally avoids medical language and diagnostic labels, instead emphasizing collaborative dialogue centered on values and strengths. How does this approach apply to children diagnosed with Attention Deficit Hyperactivity Disorder (ADHD), the most common neurodevelopmental condition? This study presents the first scoping review on this topic. A systematic search of five databases (APA PsycNET, PubMed, Web of Science, ProQuest, and Google Scholar) identified 24 records meeting the inclusion criteria. Four key insights emerged relating to the therapies: (1) philosophical foundations; (2) unique perspective on ADHD; (3) practical interventions (e.g., externalizing and separating the problem from the child, identifying ‘sparkling moments’); (4) reported effectiveness. Further research is recommended to evaluate the therapy’s empirical impact and explore its potential to empower children by co-creating a ‘health-side story’ that shifts focus from problems to strengths, relationships, and values.

## 1. Introduction

The use of diagnostic labels in child psychotherapy may prove to be a double-edged sword. On the one hand, labels can provide children and parents with a sense of meaning and control. They may also reduce parental guilt and facilitate access to valuable treatments and services. On the other hand, labels may expose children to stigma and negative self-fulfilling expectations. Over time, such labels can harm a child’s self-esteem and diminish their confidence in their ability to change or to benefit from psychotherapy [1,2,3,4]. This concern is particularly relevant to the most common childhood diagnostic label: ADHD—Attention Deficit Hyperactivity Disorder [5,6].

According to the Diagnostic and Statistical Manual of Mental Disorders (DSM-5-TR), ADHD is a neurodevelopmental condition that impairs attention and executive functions, potentially leading to a range of serious outcomes and complications [6]. Expert consortiums typically conceptualize ADHD as an inborn and lifelong condition with strong neurobiological foundations [7,8]. Correspondingly, the DSM-5-TR places ADHD within its subsection on neurodevelopmental disorders, and some experts have referred to it as ‘the diabetes of the brain’ [9]. It is, therefore, possible that some children who receive this diagnosis may internalize these medical messages and develop an entrenched perception of its inevitable consequences.

While the biomedical model of ADHD remains dominant and is accompanied by a growing body of neurobiological literature and widely adopted in clinical settings, narrative therapy offers a fundamentally different approach. Rather than focusing on presumed biological deficits, it invites clients to re-author their experiences through storytelling and meaning making. In this sense, narrative therapy represents a radical departure from conventional practice and aligns with contemporary cultural movements that emphasize pluralism, identity exploration, and resistance to diagnostic determinism [10].

In narrative therapy, clients are encouraged to question the common perception of the diagnostic label. They are also guided to *externalize* the label and distinguish it from their broader, more nuanced identity [11]. As implied by the therapy’s name and its underlying postmodern philosophy (see the Results section for more details), narrative therapists typically refer to the diagnostic label as ‘only one possible and rather thin story’ about the child [12]. In the therapist’s view, this story does not necessarily offer the most accurate or helpful representation of the child. Therefore, during therapy, children and their parents are invited to ‘re-author’ the dominant story about the ‘problem’ that brought them to seek help [13]. In this emerging narrative, clients are encouraged to highlight their strengths and psychological resources, fostering a renewed sense of optimism and self-confidence (e.g., [14,15]).

Indeed, this approach may sound promising, but can it truly support children diagnosed with neurodevelopmental conditions such as ADHD in real-world settings? What evidence currently exists regarding its effectiveness in addressing ADHD, and how does it actually work? After all, narrative therapy does not aim to reduce symptoms directly, but rather to promote a shift in how clients understand and relate to their life challenges, including the specific challenge commonly referred to as ADHD.

To date, there are no simple answers to these questions. To our knowledge, only a limited number of studies have investigated the effectiveness of narrative therapy for ADHD, and no comprehensive review has yet been published on this topic. The aim of this article is, therefore, to present the first scoping review of the scientific literature accumulated over recent decades regarding narrative therapy for ADHD. Through this review, we aim to begin addressing the questions raised above, introduce key principles and interventions of the therapy, and highlight its main strengths and limitations, which merit further exploration in future research.

## 2. Method

The present review followed the extended guidelines of the Preferred Reporting Items for Systematic Reviews and Meta-Analyses for Scoping Reviews (PRISMA-ScR) [16]. The registered protocol is available on the Open Science Framework (OSF): https://doi.org/10.17605/OSF.IO/7ZEPX (accessed on 21 May 2025). The literature search was conducted in January 2024. Below is a detailed description of the search strategy, inclusion and exclusion criteria, and methods of charting and summarizing the data.

### 2.1. Search Strategy

The search terms for narrative therapy were “Narrative Therapy” and “Narrative Approach” (to locate records referring to this therapy but using a different lingo). The search terms for ADHD were the popular acronym “ADHD” and the opening two words of the formal diagnostic term, “Attention Deficit” (to locate records that do not use the common acronym or those using diagnoses from previous editions of the DSM).

**Figure 1:**
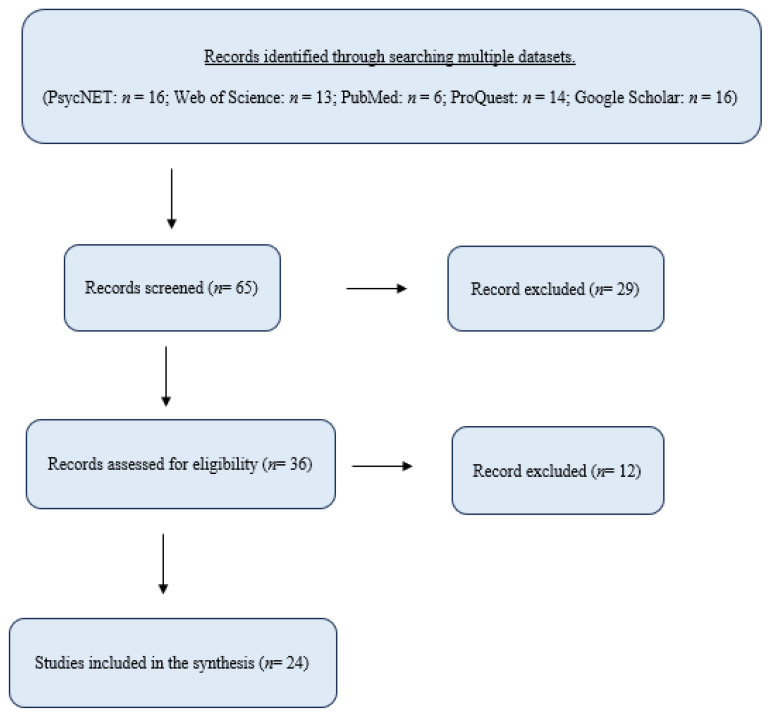
Preferred Reporting Items for Systematic Reviews and Meta-analyses for Scoping Reviews (PRISMA-ScR) Flowchart.

Figure 1 describes the search workflow. The four possible combinations of these terms (i.e., “ADHD” and “Narrative Therapy”; “ADHD” and “Narrative Approach”; “Attention Deficit” and “Narrative Therapy”; and “Attention Deficit” and “Narrative Approach”) were searched in all the available search fields of three prominent databases in psychology, medicine, and general science: APA PsycNET, PubMed, and Web of Science. A complementary search for unpublished work was conducted using the ProQuest search engine. In this search, the terms were restricted to the abstract field to avoid doctoral dissertations that do not address these terms directly. Finally, a last search was conducted using Google Scholar to locate potentially highly relevant academic articles that were not indexed in one of the previous conventional databases. This search was restricted to the title field only.

### 2.2. Inclusion and Exclusion Criteria

In light of the limited literature on narrative therapy for ADHD, this review implemented wide inclusion criteria, which comprised all types of publications and languages. This inclusive approach yielded an initial pool of 36 records after the removal of duplicates (Figure 1). The initial pool included 18 journal articles, 9 dissertations and theses, 2 books, 2 book chapters, and 5 book reviews.

Then, in the second step of the systematic search, records that did not address the topic of this review (i.e., narrative therapy for ADHD) were excluded from the final dataset. To mitigate potential bias, particularly regarding non-peer-reviewed sources, such as book chapters, this exclusion process was conducted independently by two authors. The authors read the titles and the abstracts of all the records carefully and judged their relevance to the topic. In cases where the title and the abstract did not provide sufficient information (e.g., in records of book chapters), the authors reviewed the complete manuscript. They also searched for the keyword ‘narrative’ within each manuscript to evaluate its precise meaning within its context. Notably, most of the records that were excluded in this stage were removed because the term ‘narrative’ did not refer to narrative therapy but to methodological (narrative) content analyses or to (narrative) presentations of results. Altogether, in this process, the authors agreed independently and unanimously to remove 12 records (7 journal articles and 5 dissertations) that did not address the topic at hand.

### 2.3. Methods of Charting and Summarizing the Data

The final dataset of the records that met the inclusion criteria is presented in the results section. The methods of charting and summarizing the data followed the guidance on the conduct of narrative synthesis of findings from literature reviews [17]. This methodological choice was employed in light of the characteristics of the dataset, which were not suitable for a quantitative meta-analysis. These included the following: (1) a mix of theoretical and empirical records; (2) the presence of qualitative studies; (3) an insufficient number of quantitative studies reporting interpretable outcome statistics; (4) mostly low-quality research designs with small samples.

In practice, we created a charting form in Microsoft Excel to document the essential details from each record. These included the type of the record, its methodologies, and key findings or insights (Table 1). We divided the charting of the data into three steps. In the first step, we conducted a careful reading of the theoretical literature to capture the essence of narrative therapy for ADHD, organize its key principles and insights, and identify the common themes between the collected records. Then, in the second step, we conducted a careful reading of the empirical studies to organize their findings and evaluate their quality and strength [18]. Finally, in the third step, we mapped the empirical findings and theoretical insights onto the overarching themes that emerged from the collected records (see the results).

## 3. Results

The scoping review of literature, using the PRISMA-ScR guidelines and the selection criteria described above, yielded a final set of *24 records* addressing the topic of narrative therapy for ADHD (Figure 1). These records included eleven journal articles, four doctoral dissertations, two books (of which one book included only one relevant chapter), two book chapters, and five book reviews (Table 1).

### 3.1. Characteristics of the Selected Records

Table 1 summarizes the main characteristics of the final set of 24 relevant records, including their key methods and findings/insights. A total of 11 theoretical records and 13 empirical records were published between the years 1996 and 2023. Most of the collected records were published in North America, but a significant number of the empirical studies were conducted in Iran (*n* = 7). Of the theoretical records, two titles are specifically noteworthy since they offer a direct and holistic description of the topic at hand: the book chapter “From deficits to special abilities: Working narratively with children labeled ADHD” [19] and the book “Treating Huckleberry Finn: A new narrative Approach to working with kids diagnosed ADD/ADHD” [12]. Correspondingly, four of the five book reviews that emerged in the systematic search addressed this last book by Nylund.

Apparently, the fifth book review addressed a well-known book about narrative therapy, which was written by one of its founders [14]. This book, by David White, provides ‘maps of narrative practice’ that extend the scope of the issue at hand, and it did not appear in the current systematic search. However, since some of its content does relate specifically to applications of the therapy for children diagnosed with ADHD, we refer to this book as well in the detailed presentation of the results below.

### 3.2. Synthesis of the Results

The charting and summarizing of the data from the entire set of records yielded four overarching themes relating to the therapies: (1) philosophical foundations; (2) unique views about ADHD; (3) main interventions and strategies; (4) reported effectiveness. The sub-sections below present in detail the key findings and insights of this review for each one of these themes. Representative references to the collected records of this review are embedded throughout these sub-sections to direct readers to further information about the specific aspects of narrative therapy that are presented in each theme. Complemented information about the findings and insights of this review is also available in Table 1.

#### 3.2.1. Philosophical Foundations

Many of the collected records in this review discuss the philosophical underpinnings of narrative therapy. In this section, we briefly review the key theoretical foundations that have shaped this approach.

##### Language, Meaning, and Power in Postmodern Thought

Narrative therapy evolved from postmodern critical theories, particularly those centered on discourse, knowledge, and power dynamics (e.g., [15,20]). According to these theories, language is not just a neutral tool for communication; it is a social construct that reflects and reinforces cultural norms and values, shaping the way individuals make meaning of their experiences. This view applies even to seemingly objective terms such as ‘human nature’, ‘scientific truth’, or ‘medical diagnosis’. Prominent postmodern thinkers such as Michel Foucault argued that our social systems, including those aimed at promoting justice, science, or mental health, are structured by asymmetric power relations that can marginalize those who deviate from societal norms.

##### Clinical Hierarchies and the Risk of Labeling

Foucault’s critique of medicine and psychiatry suggested that scientific language can be used, even unintentionally, to classify and control individuals by labeling those who differ from the norm as disordered or deficient. This perspective does not imply that clinicians or scientists are driven by a desire to dominate others but rather highlights the need to be aware of how language and institutional structures can reinforce hierarchical dynamics. For example, terms such as ‘non-compliant patient’ may reflect and perpetuate an implicit power imbalance between clinician and client (e.g., [15,21]).

##### Reimagining the Therapeutic Relationship

Inspired by these postmodern insights, the founders of narrative therapy, Michael White and David Epston, emphasized a collaborative, respectful relationship between therapist and client. While many therapeutic approaches value empathy and trust, narrative therapy takes this further by rejecting the idea that therapists ‘own the knowledge’. Instead, clients are seen as the experts of their own lives. White described the therapist’s role as akin to that of an ‘investigative reporter’, approaching the client’s story with genuine curiosity and openness [14].

##### Language, Identity, and the Power of Re-Authoring

Narrative therapists are especially attuned to the influence of language on clients’ identities. They often interpret the stories clients tell about themselves as shaped by dominant cultural narratives, which dictate what is considered normal or valuable. Rather than fitting clients’ experiences into pre-existing diagnostic templates, narrative therapists prefer ‘experience-near’ descriptions that stay close to the client’s own words and meanings. They also emphasize the broader social context in which problems occur, seeing life difficulties as arising from tensions between the individual and their environment—not from individual pathology alone. Accordingly, they view meaningful change as something that involves not only the child but also their caregivers, peers, and community (e.g., [15,19]).

Even in situations where clients cannot change their external circumstances, narrative therapy suggests that recognizing the social forces acting upon them can have therapeutic value. By understanding that they are not necessarily the root cause of their problems, clients may experience a reduction in shame and guilt. This, in turn, can empower them to challenge dominant biomedical interpretations and to re-author their personal narratives with a sense of competence, self-compassion, and hope, as discussed in Section 3.2.3.

#### 3.2.2. View of ADHD

Some of the records collected in this review derive their research motivation from the more specific field of critical psychiatry. In order to avoid the potential harms of the diagnostic label (see the opening of this article), clients of narrative therapy are invited to rethink their beliefs about ADHD. This process is sometimes called the ‘deconstruction of ADHD’, and its goal is to soften the potentially deterministic and discouraging messages embedded in the diagnosis (e.g., [20,22]).

Assuming the aforementioned egalitarian and respectful therapeutic relationship, narrative therapists do not rush to undermine the preconceptions of clients. They listen to what they call ‘the deficit-saturated story’ with great attention and compassion and acknowledge the stigma and the agony that surround the diagnosis (e.g., [23,24]). Often, they begin asking about this story using questions about its potential benefits for the clients. For example, therapists may inquire about the sense of meaning and control that ‘comes with the diagnosis’ or about the common belief that ‘if the medication works, it proves that the diagnosis is real’ [24]. In this way, clients can feel understood while beginning to realize that even medical terms can be ‘stories’ when it comes to mental health, as implied by the underlying postmodern philosophy of the treatment (see Section 3.2.1).

Then, in the next phase of the conversation about the diagnosis, therapists may start deconstructing its common meaning and wonder whether it contributed to the ‘silencing of the authentic voice’ of their clients (e.g., [21]) and to their low self-esteem through the self-fulfilling prophecy described in the opening of this article. Therapists may also ask parents (and teachers) whether they started viewing many of the child’s behaviors through the prism of this diagnostic label of ADHD [12]. Their working assumption is that the organizing ‘ADHD story’ may cause people to ‘code’ many of the child’s behaviors as evidence for the existence of the diagnosis while missing other behaviors that do not fit this organizing story (e.g., the times when the child reads quietly or behaves kindly).

In addition, therapists can ask parents about the emotional cost of the diagnostic label and its treatment of choice with medication. For example, they might ask: Can high expectations from medication lead to long-term disappointment? Can daily use of a ‘medicine’ give children the impression that they suffer from an illness? Might regular reliance on medication lead children to believe they are incapable of functioning without it to the point of developing psychological dependence?

Importantly, these questions do not disregard families’ desire for clarity, guidance, or even medical support. Narrative therapists are encouraged to meet such needs with openness and curiosity rather than opposition. While the diagnostic label is examined critically, its psychological and practical value for families is also acknowledged. In this way, narrative therapy does not aim to discredit medical choices but rather to support families in exploring how such choices fit within their broader narratives, values, and goals.

Finally, therapists may ask parents about the social forces and power dynamics that are involved in the discourse about ADHD. For example, therapists may ask parents about the role of the educational system in the creation and maintenance of ADHD. They can also inquire about the clients’ experiences with authoritative figures and whether they felt they were pushed into compliance [24]. In the same vein, therapists may ask parents if they received comprehensive information about the benefits and risks of the medications and whether they feel that they could give their full and authentic ‘informed consent’. Such questions are assumed to empower and ‘give voice’ to clients who sometimes feel oppressed or silenced (e.g., [20,21]). Through this deconstructing conversation, clients can become aware of the complex societal factors that are involved in the diagnostic process of ADHD, be inspired to resist the pressure exerted on them, and become the ‘heroes’ of their life stories.

#### 3.2.3. Key Interventions and Strategies

But how does narrative therapy work in practice? The assembled records of this review portray a rich corpus of interventions and strategies aimed at helping clients ‘re-write their story’ (Section 3.2.2). In this review, we documented four leading interventions and strategies, which are outlined below in the following sub-sections of chapter 3.2.3.

##### Externalizing and Separating the Problem from the Child

Probably the most recognized intervention of narrative therapy is the externalizing conversations (e.g., [12,14,23]). In order to cope with the ‘deficit-saturated story’ that brought the parents to seek therapy (e.g., “our children are ADHD; they drive everyone crazy”), narrative therapists aim to externalize the problem and separate it from the client. In this way, “the problem becomes the problem, not the person” [14] (p. 9). In narrative therapy, a child cannot ‘be’ ADHD. At most, he or she can face a challenge we call ADHD. Although seemingly minor, this semantic shift can be powerful within a postmodern narrative framework that emphasizes the influence of language. During the externalizing conversations, the problem can be given a comical name by the child and the therapist. Nylund, for example, mentions ‘Giggles’ and ‘Trouble’ as names used to describe ADHD in the therapy [12]. Eventually, this externalization conversation allows clients and the therapist to inspect the problem together as if it were lying in front of them on an operating table.

Notably, the reviewed records typically refrained from viewing the problem as a binary entity. Instead of trying to determine whether the child has ADHD or not, narrative therapists often deliver a message that multiple stories can be told to describe the problem, depending on the different perspectives of the storytellers (i.e., the parents, the teachers, and the children themselves). This message also serves the externalization process because it implies that the problem may not be located within the child only. The problem is typically conceptualized as context-dependent and transient. It may come and go in different times and settings, and therefore, it requires a range of accommodations, including macro changes in the environment, rather than just micro changes in the child’s behaviors (e.g., [15,21]).

To this end, narrative therapists invite family members and teachers to share their unique perspectives about the problem and about the child. Caregivers are encouraged to use ‘experience-near’ language, replacing clinical terminology (e.g., hyperactive, distracted, or uninhibited) with more neutral or even positive language (e.g., energetic, spirited, enthusiastic, or alert). In this way, therapists aim to normalize age-appropriate boisterous behaviors and soften polarized narratives that imply that the child is ‘bad’ or ‘ill’ (e.g., [22]). Still, if clients are comfortable using the specific term of ADHD, therapists will not insist on removing this term from the discourse. Instead, they will focus on using externalized language and flagging potentially deterministic expressions, such as: “Jonny is ADHD; he has a chronic deficit in his brain”.

##### Integrating Playful Communication and Metaphors

Appropriate language is often the main road to externalization. However, young children might feel alienated in a discourse that relies heavily on language. Thus, narrative therapy for children often incorporates a range of playful communication strategies, such as role-plays, arts and crafts, and fun games. Using dolls, teddy bears, or superheroes, narrative therapists strive to ‘meet children where they are’ and ‘speak their language’ (e.g., [19,25]). Young children can be invited to draw the problem, sculpt it in sugar dough, or paste newspaper clippings to represent it. Older children can be invited to write a letter to the problem (e.g., [23]). Not only is such a playful-artistic discourse more suitable for children, but it is also particularly suitable for children diagnosed with ADHD who often possess compatible traits, such as high-energy, fruitful imagination, and creativity (for further details, see Section 4.3).

In addition, as can be expected from a therapy that emphasizes storytelling, narrative therapy embraces the usage of metaphors. Metaphors are thought to bridge language gaps that exist between children and adults, but they, too, are thought to serve the externalization process (e.g., [23]). The usage of metaphorical language encourages clients to separate themselves from the problem and maintain a kind of ‘relationship’ with it (e.g., [15]). A metaphor, for example, may be a lightsaber that represents the child’s will of power, which can help them ‘defeat the problem’ as if it was ‘a foreign enemy’.

Alternatively, the lightsaber can be used to ‘clear the road’ for solutions that would make it possible to live peacefully alongside the problem and maybe even grow to appreciate its advantages (see Section 4.3). In narrative therapy, a child can become a ‘monster trainer’ or a ‘ninja of the mind’ that can choose when to stay focused and sharp like a knife and when to be fast and furious like a Ferrari. This may be especially effective with diagnosed children who already think of themselves as having ‘an extra factor in their brain that makes it go quicker’ [24]. In general, such metaphors from the field of martial arts are especially suitable for ADHD because they encourage focus and self-discipline as well as acceptance and willingness to live alongside the problem [19].

##### Identifying and Celebrating Unique Outcomes (‘Sparkling Moments’)

Another distinctive feature of narrative therapy, according to the reviewed records, is the active search for behaviors and traits that do not align with the deficit-saturated story of the clients (e.g., [14,15]). These behaviors and traits are called *unique outcomes*, or, more figuratively, *sparkling moments*, and their role is to enrich the clients’ preliminary perspective about their difficulties and facilitate the creation of a more hopeful and empowering story. Such sparkling moments can be the times when a child is reading a book calmly or helping out with the household chores.

Sparkling moments are typically cherished and celebrated during the therapy and serve as entry points through which the deficit-saturated story can be enriched (e.g., [21]). According to the narrative approach, the more the story ‘gets richer’, the more the parents are expected to discover the ‘poverty’ of the diagnostic label; that is, its inability to describe the round and wonderful character of their child. In addition, the more the story incorporates a sympathetic and admiring view of the child, the higher the chances are that the child’s self-image will be restored.

This process of identifying and amplifying unique outcomes is central to the overarching goal of narrative therapy: to empower the child and their family. In this context, *empowerment* refers to helping clients reclaim a sense of agency, dignity, and optimism by highlighting their values, personal strengths, and previously overlooked moments of competence. Rather than focusing solely on symptom reduction, empowerment in narrative therapy reflects a shift in self-understanding—from being defined by a problem to becoming the active author of one’s life story.

In a way, the therapist’s role is to help clients ‘connect the [positive] dots’ and become active in the re-writing of their life story (e.g., [21]). To this end, parents are called to articulate their children’s strengths—a simple intervention that also fosters warmer parental feelings toward the child (e.g., [22]). Similarly, children may be given concrete assignments to collect favorite memories, values, identities, and positive qualities (e.g., [20]), perhaps using photos or personal diaries. Children may also be guided to visualize themselves at their best through relaxation techniques and role plays (e.g., [23]). Altogether, in the new story that is being written in the therapy, the hero (i.e., the child) is no longer ‘just a kid dealing nicely with a problem’. The hero becomes a round and inspiring character with a promising future and multiple strengths (for further details, see Section 4.3).

##### Internalizing the New (Hopeful and Empowering) Story Being Written in the Therapy

To make sure that clients will ‘believe’ and internalize the hopeful and empowering story, the reviewed records suggest using a range of complementing ‘story aids’ such as letters, ceremonies, and real audiences (e.g., [25]). For example, therapists may write or record short messages to their clients, reminding them to implement the metaphors and strategies they were discussing in the sessions. In these messages, therapists may say something like: “Jonny, you got it. Don’t forget to use your ninja powers today during math class so Giggles will not start again with his tricks and shticks”.

In the same vein, therapists may recruit witnesses and fans—family members, friends, and teachers who can ‘bring the story to life’ by being its witnesses. Like problems (which are thought to derive their vividness from the social environment), good solutions need to be backed by authentic messages from the child’s surroundings. In order for Jonny to ‘really’ feel that he is ‘not just an ADHD child, but much more than that’, he must see it in his friends’ and caregivers’ eyes. In this way, the family, the school, and the community of the child become essential therapeutic and empowering resources, and the burden of change is not placed on the child only (e.g., [21]). In fact, parents and teachers are expected to be involved in the re-writing of the child’s story, and therapists may invite them to witness and celebrate the child’s success (preferably with prizes or medals). These ritualistic acts are assumed to give children an empowering feeling that they have a crowd of fans who believe in them and admire their achievements.

In addition, as part of the therapists’ efforts to extend the therapy’s discourse beyond the four walls of the clinic, therapists may also initiate strategic meetings with multiple attendees (i.e., parents, teachers, counselors, and other caregivers). In these meetings, the attendees are invited to share their impressions of the child (while refraining from using clinical language as explained above) and provide constructive suggestions that would contribute to the child’s well-being. Importantly, in this discourse, the parents are not excluded or overlooked. On the contrary, parents’ voices are cherished as they are considered the ‘experts’ when it comes to their own children (see Section 3.2.1).

The ending of narrative therapy is a cause for celebration. The therapist can give clients a certificate of excellence and even ask them for permission to share their triumph story with other clients, who could be inspired by their experience [19,25]. This intervention can be highly empowering for many children. Not only did they manage to overcome difficulties and feel more competent, but they could even teach other children how to be ninjas of the mind. They become ninja masters.

#### 3.2.4. Reported Effectiveness

While the above sections on the philosophy and interventions of narrative therapy may sound promising, the empirical evidence for its effectiveness in ADHD remains limited. Our systematic search yielded only 13 empirical studies, of which two were qualitative. The remaining nine quantitative studies did report findings indicating that narrative therapy may reduce ADHD symptoms and improve general psychological health (e.g., [26,27,28]). However, a substantial number of these studies were conducted within a single geographical context (i.e., Iran), which may limit the generalizability of the findings. In addition, most of the quantitative studies were quasi-experiments with very small sample sizes (Table 1). Based on these parameters, the overall quality of the research was judged to be low [18], in line with key dimensions commonly considered in evidence appraisal frameworks.

The only exception is the relatively new study by Karibwende and colleagues [23], which comprised a more formal, Randomized Controlled Trial (RCT). This RCT investigated a sample of 72 children and reported a large effect size (*Cohen’s d* = 1.6) with regard to the potential influence of narrative therapy on ADHD symptoms. It is also noted that this article by Karibwende and colleagues provides an informative table that outlines the content of each one of the 10 narrative therapy sessions that were implemented during the RCT. The table of sessions, alongside the aforementioned seminal texts by Nylund, complements the key interventions and strategies presented in the current review (Section 3.2.3). A further discussion of the (limited and complex) effectiveness of narrative therapy in ADHD is provided below (Section 4.1).

## 4. Discussion

Narrative therapy has been suggested as a revolutionary approach to the treatment of ADHD—a common disorder among children, which is typically perceived as a chronic neurodevelopmental deficit [6,7]. The current article provides a first scoping review of the literature that has been written directly on this topic of narrative therapy for ADHD. Following the PRISMA-ScR guidelines, this review yielded a set of 24 relevant records. The findings and insights from these 24 records are summarized in Table 1 and are mapped onto four themes, as mentioned in the results section. The first two themes included the following: (1) the philosophical underpinnings of the therapy; (2) its unconventional views about ADHD. The last two themes included the following: (3) the key interventions and strategies of the therapy; (4) its reported effectiveness.

From a practical point of view, Section 3.2.3 of the results outlines four leading and relatively distinctive interventions and strategies of narrative therapy: (a) externalizing and separating the problem from the child; (b) integrating playful communication and metaphors; (c) identifying and celebrating unique outcomes; (d) internalizing the new story being written in the therapy. From an empirical point of view, however, the robustness and the quality of the evidence supporting the effectiveness of the therapy is not satisfactory, as mentioned above (Section 3.2.4). We discuss this empirical gap next.

### 4.1. Limitations and Controversy

Despite the fact that all the documented quantitative studies addressing the effectiveness of narrative therapy for ADHD reported favorable findings, the number of these studies (n = 9) and their scientific quality were considerably low. Most of the studies were defined by their authors as quasi-experiments, while only one study met the criteria for a Randomized Control Trial [23]. This status of the available evidence places a central limitation on the literature on narrative therapy for ADHD, and further empirical research is, therefore, crucially needed.

Further empirical research is also needed in light of the controversy over the legitimacy of narrative therapy for ADHD. A major point of controversy revolves around the idea of Huckleberry Finn as a literary character that can help us understand ADHD-type children. As mentioned above, the main book on narrative therapy for ADHD by David Nylund received the heading: ‘Treating Huckleberry Finn’ [12]. Meanwhile, the International Consensus Statement about ADHD, which was issued two years later, states the following: “to publish stories that ADHD is a fictitious disorder or merely a conflict between today’s Huckleberry Finns and their caregivers is tantamount to declaring the earth flat” [8] (p. 90). In other words, while the book by Nylund compares ADHD-type children to this famous literary character of Huckelberry to remind us of his advantageous personality traits (e.g., independency, nonconformity, conscientious, resourcefulness, loyalty, and youthful innocence) and to echo the original social criticism embedded in Mark Twain’s book, others insist that this comparison is misleading. In fact, 3 of the 4 book reviews addressing the book by Nylund (Table 1) accuse narrative theorists of spreading unreliable information about ADHD and its pharmacological treatments [29,30,31].

The comparison to Huckleberry Finn is one such metaphorical lens intended not to deny the challenges associated with ADHD but to highlight the potential for viewing these behaviors in a less pathologizing light. We acknowledge, however, that this metaphor is debated and may risk romanticizing complex realities. Its value, therefore, may lie less in literal comparison and more in prompting critical reflection about how diagnostic labels shape therapeutic narratives.

The discussion around the Huckleberry Finn metaphor reflects a broader controversy surrounding narrative therapy in the context of ADHD. The current article does not presume to resolve this debate, nor does it underestimate the strong empirical limitation mentioned above regarding its reported effectiveness. However, it should be mentioned here that the very philosophy of narrative therapy questions the relevance of conventional trials to this treatment. Narrative therapy, as introduced above, does not accept the bio-medical terminology regarding ADHD. This means that ‘reducing symptoms of ADHD’ may not necessarily be a direct goal of this therapy.

At the same time, narrative therapy does not conflict with other non-pharmacological interventions designed to reduce symptoms directly, such as behavioral therapy, parent training, neurofeedback, or cognitive training [32]. These evidence-based approaches offer valuable tools and structured strategies for children and parents. Narrative therapy can complement such interventions by offering a broader conceptual lens—one that centers the child’s identity, values, and social context. In this way, it may help families make use of diverse treatments without automatically adopting deficit-based language or biomedical assumptions. Rather than viewing narrative therapy and other approaches as mutually exclusive, they can be understood as potentially synergistic elements within a holistic, empowering process.

A distinguishing feature that may set narrative therapy apart from other non-pharmacological interventions is its ambivalent stance toward symptom-based measurement. While many established approaches emphasize quantifiable symptom reduction, narrative therapy is grounded in a postmodern framework that questions the assumptions underlying such metrics and seeks to broaden the conversation beyond behavioral outcomes (Section 3.2.1). Thus, asking children to participate in a study that aims to show how therapy improves children’s behaviors may feel inappropriate for some narrative therapists. After all, the goal of the therapy is essentially to help the child and the family ‘free themselves’ from the traditional bio-medical story and construct their own ways to thrive in the face of their life challenges. These (arguably vague and complex) goals may be difficult to quantify and evaluate within the context of traditional controlled trials.

### 4.2. Future Research Directions

Given the limitations of the existing empirical evidence and the philosophical distinctiveness of narrative therapy, future research should explore alternative methodological approaches that move beyond standardized, symptom-based metrics. These traditional measures may not fully capture the therapeutic changes that narrative therapy seeks to facilitate, particularly those related to identity, meaning making, and social context.

Researchers are therefore encouraged to incorporate outcome indicators that better reflect the narrative framework, such as client satisfaction, the depth and richness of narrative transformation, the extent of externalization, levels of family engagement, and reductions in stigma or self-stigmatizing beliefs. Mixed-methods designs and narrative outcome analysis may be especially suitable, allowing for both structured measurement and in-depth exploration of clients’ evolving self-narratives.

Additionally, research efforts should seek to address the gap in high-quality, well-powered clinical trials. While RCTs may present philosophical tensions with narrative therapy’s epistemology, carefully designed studies that respect client agency and contextual variation could still provide meaningful insights. For example, future trials might combine flexible, participant-driven interventions with both qualitative interviews and adapted outcome metrics.

Finally, researchers are advised to remain attentive to the conceptual assumptions embedded in their measurement tools. The routine use of symptom-based outcome measures, such as the Conners’ Parent Rating Scale [33], may be seen as inconsistent with the therapeutic stance of narrative work. As described earlier, the ‘symptoms’ of hyperactivity, distraction, and impulsivity are not necessarily viewed in narrative therapy as deficits to be corrected, but rather as behaviors that can be re-interpreted in a more contextual and empowering light. In many cases, these behaviors are re-framed as neutral or even positive traits, such as creativity, spontaneity, or nonconformity, as implied by the metaphorical comparison to Huckleberry Finn. This perspective is not intended to idealize or deny the difficulties associated with ADHD but to expand the interpretive space for clients and therapists alike. The next section is dedicated to a theoretical and empirical exploration of this core idea.

### 4.3. Potential Justifications for the Positive Reframing of ADHD

Critics of narrative therapy might argue that the suggested positive view about ADHD, which is held in the therapy, is detached from the child’s reality. After all, multiple studies demonstrated the harms of ADHD throughout the person’s life [7,34]. Nevertheless, it should be noted here that the ADHD literature also contains a stream of articles and books that are intrigued by the potential ‘bright side of ADHD’ (e.g., [35,36]). This stream extends far beyond the literature on narrative therapy, as positive aspects of the disorder are being studied even by researchers who support the diagnosis (e.g., [37]).

Some scholars have argued, for example, that the distractibility and impulsivity tendencies characterizing ADHD can take a positive form of courage, cognitive flexibility, fortitude, and resilience [38]. Others contended that individuals with ADHD are able to consider multiple aspects of a problem in a kind of a ‘lateral thinking that opens things up to big ideas, albeit in jumbled succession’ [39] (pp. 128–129). Apparently, even the impulsivity and risk-taking qualities typically associated with ADHD are thought by some scholars to be valuable in certain highly challenging contexts such as firefighting, construction work, and the military [40,41,42,43].

#### 4.3.1. Potential Advantages of ADHD from Evolutionary Psychology Perspective

A plausible theoretical explanation for this positive view of ADHD derives from evolutionary psychology. Hartmann, for example, theorizes that individuals with ADHD-like traits can be thought of as ‘hunters in a farmer world’ [44]. In his view, risk taking and impulsivity were probably considered in historical hunter-gatherer societies as virtuous personality strengths. ADHD-type ‘hunters’ are hypothesized to be the ones to monitor the environment for food and threats (distractibility) and to engage in abrupt, high-risk actions without hesitation (impulsivity/risk taking). Essentially, this evolutionary-inspired point of view postulates that ADHD-like traits were preserved during the natural selection process because they contribute to the survivability of the person or of the species in certain environments, such as battles or natural disasters [45,46].

Empirical support for this hypothesis may be found in the study by Grossman and colleagues [47], who applied the well-researched ‘gorilla task’ among college students with and without a diagnosis of ADHD. The gorilla task is a known research methodology in which participants are instructed to monitor a video showing ball-passing between a circle of players. Typically, many participants are so caught up with the task, to the point that they miss (i.e., demonstrate an inattentional blindness to) prominent stimuli, such as a walking-by gorilla [48]. Apparently, participants diagnosed with ADHD were significantly more aware of the ‘unattended stimuli’ than participants without ADHD. ADHD participants were better at detecting a gorilla figure that entered the circle of players while beating her chest and were better at noticing another typically unattended stimulus in which one of the ball players left the scene [47]. According to the authors of this study, their results support Hartmann’s hypothesis that individuals with ADHD have attentional advantages of hunters who are able to perceive information in a simultaneous manner from both attended and unattended channels.

Indeed, modern society seems to be less tolerant of ADHD-like characteristics despite their alleged evolutionary value. However, some occupational environments, as mentioned above, may benefit significantly from these ‘hunter’ traits. A line of research led by Johan Wiklund, for example, ties the impulsivity and hyperactivity components of ADHD to entrepreneurship and the probability of starting a business [49,50]. A large study of over 10,000 students suggested that this relationship between ADHD-like behaviors and entrepreneurial intentions is partially mediated by the aforementioned tendency of risk taking [51]. Further examination of this dataset determined that the link between ADHD symptoms and entrepreneurship remained even when the level of the symptoms met the diagnostic threshold required for a clinical diagnosis of ADHD [52]. This fit between characteristics of ADHD and the business/entrepreneurship world may be explained by the entrepreneurship environment that, in some cases, appreciates speed over accuracy. Mainstream school environments, in contrast, do not seem to fit the sensation-seeking tendency of ADHD-type children [32]. ADHD, from this perspective, can be conceptualized essentially as an ‘evolutionary mismatch’ [45].

#### 4.3.2. Potential Advantages of ADHD from Cognitive Neuroscience Perspective

Critics of narrative therapy may still argue that these advantages are far less prevalent than the problematic aspects of ADHD. However, they would probably not deny the observed tendency of ADHD-type individuals to *creativity*. Although the existing literature on ADHD and creativity produced mixed results, the link between (sub-clinical) symptoms of ADHD and creativity seems to be acknowledged by scholars across camps. Hoogman and colleagues [37], for example, proposed that ADHD and creativity share some identical brain mechanisms. It is possible, then, that in some settings, the lessened cognitive control and lower inhibitions characterizing ADHD are translated to divergent thinking—a cognitive pattern that allows the person to explore multiple possibilities, produce original connections between distant concepts, and generate a flow of creative, even if not always practical, ideas [35,53,54].

A potential theoretical explanation for this link between ADHD and creativity has been suggested recently by Silberstein and colleagues [55]. In their view, the increased creativity characterizing ADHD-type individuals originates from the reduced inhibition of their *brain’s default mode network*—the ‘resting mode’ of the brain, during which the person is not engaged in a specific, cognitively demanding task. This resting mode (e.g., during long drives, showers, or sports activities) allows the human mind to wander freely, explore remote connections between concepts, and sometimes produce unexpected ideas and solutions. Correspondingly, a primary characteristic of Eureka experiences (sometimes called Aha! Moments) is their suddenness [56]. When people disengage from a problem and allow themselves to ‘just wonder’, utilizing their brain’s resting-state activity, a solution or insight might suddenly appear, allowing them to present ideas ‘outside of the box’ [57].

**Table 1:** Detailed characteristics of the 24 records that met the inclusion criteria of the current review.

Authors (Year)	Title	Document Type, Publisher, and Language	Methods	Key Findings/Insights
Dallos & Vetere, 2022 [22]	Systemic therapy and attachment narratives: Applications in a range of clinical settings. (See specifically Chapter 5)	Book, Routledge, English	This book explores how attachment-based ideas can be used in clinical practice. Chapter 5 of this book is dedicated to therapeutic work with children diagnosed with Autism Spectrum Disorder and ADHD. This work is inspired by the narrative approach.	Key recommendations to therapists are as follows: To implement less medical terminology (e.g., energetic, spirited, enthusiastic, and alert instead of hyperactive, distracted, and uninhibited); normalize age-appropriate boisterous behaviors; soften polarized narratives that see the child as ill or bad; use externalized language and move toward less medical and problem saturated narratives; and develop narratives that emphasize the child’s strengths and competencies, thus fostering warmer feelings from their caregivers.
Darvish Damavandi et al., 2020 [58]	The effectiveness of narrative therapy based on daily executive functioning and on improve the cognitive emotion regulation in children with attention deficit/hyperactivity disorder	Journal Article, Journal of Psychological Science, Persian	Quasi-experiment in which children diagnosed with ADHD (aged 9–11) were allocated to narrative therapy with executive functions training.	Narrative therapy with executive functions training decreased maladaptive strategies and increased adaptive and emotional cognitive regulatory strategies.
Edwards, 2022 [20]	Using a Narrative Approach to Explore Identity with Adolescents Who Have Been Given a Diagnosis of Adhd	Dissertation, University of Sheffield, English	Qualitative research among four adolescents diagnosed with ADHD. The research consisted of 4 narrative-oriented sessions with each adolescent, and the leading narrative method was ‘the bicycle of life’. The goals of these sessions were to explore the adolescents’ strengths and difficulties as well as their perceptions about their diagnosis and its effects on their sense of self. These sessions are also intended to empower the adolescents.	Narrative-oriented sessions privileged the adolescents’ authentic voice and encouraged them to identify their strengths and values (e.g., having a sense of humor, being a keen sportsman, and being an animal lover).
Emadian et al., 2016a [59]	Effects of Narrative Therapy and Computer-Assisted Cognitive Rehabilitation on the Reduction of ADHD Symptoms in Children	Journal Article, Journal of Babol University Of Medical Sciences, English (Iran)	Quasi-experiment in which 30 children (aged 7–12) were allocated to narrative therapy (8 sessions), computer-assisted cognitive rehabilitation (10 sessions), and control (no treatment).	Narrative therapy (and computer-assisted cognitive rehabilitation) was effective in reducing ADHD symptoms.
Emadian et al., 2016b [60]	Comparing the Effectiveness of Behavioral Management Training in Parents and Narrative Therapy in Children with Attention Deficit Hyperactivity Disorder on Quality of Mother-Child Relationship	Journal Article, Journal of Mazandaran University of Medical Sciences, Persian	Quasi-experiment in which 30 children diagnosed with ADHD (aged 7–12) and their mothers were allocated to group narrative therapy (8 sessions), behavioral management training by Barkley (9 sessions), and control (no treatment).	Both narrative therapy and behavioral management improved the quality of mother-child relationships compared to controls. No significant differences were observed between the two types of treatments.
Hamkins, 2008 [61]	Maps of Narrative Practice: [Review]	Book Review, Psychiatric Services, English	Review of the book “Maps of Narrative Practice” by Michael White.	In this positive book review, the author praises the content and structure of ‘this brilliant new book’ by White. The book is said to be ‘an important text by a master’. Overall, the narrative approach, which is presented in a ‘rigorous and graceful’ manner in the book, strives to move clients ‘from a problem-saturated identity to a value-based identity that supports actions’. In this way, clients can ‘free themselves from their problems’.
Han et al., 2015 [62]	The Effects of Narrative Therapy for Children with Attention Deficit Hyperactivity Disorder	Journal Article, Journal of Korean Neuropsychiatric Association, Korean	Quasi experiment in which 16 children diagnosed with ADHD were allocated to narrative therapy (6 weeks of medications and 11 sessions of narrative therapy) and medication treatment (6 weeks of medications and education for behavior controls).	Narrative therapy improved respect for children in parent-child interactions and children’s self-control (compared to the medication group).
Hosseinnezhad et al., 2020 [63]	Comparison of the Effectiveness of Anger Management Training based on Cognitive Behavioral Therapy Approach and Narrative Therapy on Academic Self- efficiency and Academic Resilience in Students with Attention Deficit/Hyperactivity Disorder (ADHD)	Journal Article, Quarterly journal of child mental health, Persian	Quasi-experiment in which 30 boys diagnosed with ADHD (aged 11–12) were allocated to narrative therapy (8 sessions), anger management training (12 sessions), and control (no treatment).	Narrative therapy improved academic self-efficacy by the end of the treatment and in a 2-month follow-up (compared even to anger management).
Jacobs, 2001 [29]	Treating Huckleberry Finn: A new narrative approach to working with kids diagnosed ADD/ADHD.	Book Review, American Journal of Psychotherapy, English	Review of the book “Treating Huckleberry Finn: A New Narrative Approach Working with Kids Diagnosed ADD/ADHD” by David Nylund.	Although the author finds value in the clinical part of the book (which is said to reflect ‘a very compassionate, engaging, and innovative approach’), the book review is mostly negative. The book, according to this reviewer, provides an unjust criticism of the diagnosis and romanticizes the problem, and its author (Nylund) is accused of ignoring scientific evidence, contradicting himself, politicizing the discourse, using scare tactics, and providing misinformation.
Jenkins, 2001 [30]	Treating Huckleberry Finn: A new narrative approach to working with kids diagnosed ADD/ADHD.	Book Review, Psychiatric Services, English	Review of the book “Treating Huckleberry Finn: A New Narrative Approach Working with Kids Diagnosed ADD/ADHD” by David Nylund.	The author recognizes ‘some interesting and useful therapy techniques’ in the book by Nylund, but the book review is completely negative. The book, according to this reviewer, “contains logical errors and specious arguments, and it misrepresents current scientific and medical practice”. It also lacks convincing evidence that could support the efficacy of the proposed treatment. Correspondingly, its author (Nylund) is accused of promoting a conspiracy without evidence and repeating ‘tired old claims’.
Karibwende et al., 2023 [23]	Efficacy of narrative therapy for orphan and abandoned children with anxiety and attention deficit and hyperactivity disorders in Rwanda: A randomized controlled trial.	Journal Article, Journal of Behavior Therapy and Experimental Psychiatry, English	Randomized controlled trial in which 72 children (mean age = 10) were allocated to group narrative therapy (10 sessions, 12 children in a group) and control (waiting list).	Narrative therapy was found to be effective for ADHD with large effect size. The article also provides a table with detailed descriptions of each one of the 10 sessions of the therapy.
Looyeh et al., 2012 [26]	An exploratory study of the effectiveness of group narrative therapy on the school behavior of girls with attention-deficit/hyperactivity symptoms.	Journal Article, Archives of Psychiatric Nursing, English (Iran)	Exploratory study in which 14 girls (aged 9–11) were allocated to narrative therapy (12 sessions) and control (waiting list).	Narrative therapy had a significant effect on reducing ADHD symptoms 1 week after completion of treatment. The effect was sustained after 30 days.
Mancuso & Yelich, 2003 [64]	Review of Treating Huckleberry Finn: A Narrative Approach To Working With Kids Diagnosed ADD/ADHD.	Book Review, Journal of Constructivist Psychology, English	Review of the book “Treating Huckleberry Finn: A New Narrative Approach Working with Kids Diagnosed ADD/ADHD” by David Nylund.	This in-depth book review endorses Nylund’s work and positions it within the field of constructivist psychology. The authors explain that the book corroborates and complements their previous work on the ‘inadequacy’ of the diagnostic label of ADHD and present its key therapeutic steps in detail.
Moradian et al., 2014 [28]	The effectiveness of narrative therapy based on executive functions on the improvement of inhibition and planning/organizing performance of students with ADHD	Journal Article, Journal of School Psychology and Institutions, Persian	Quasi-experiment in which 20 boys diagnosed with ADHD (aged 7–10) were allocated to narrative therapy, which incorporated executive functions training (14 sessions) and control (waiting list).	Narrative therapy with executive functions training improved inhibition and planning/organizing behaviors.
Morrison, 2020 [15]	Speaking ourselves quickly into meaning: social work praxis, narrative therapy, and women with ADHD	Dissertation, University of the Fraser Valley, English	Thematic literature review of the following search terms: ‘women ADHD’, ‘narrative therapy ADHD’, ‘narrative therapy social justice’, ‘social justice ADHD’, ‘social construction ADHD’, ‘feminism and narrative therapy’, and ‘narrative therapy praxis’. ADHD-related literature was confined to records published since 2010. Narrative therapy-related literature was confined to records published since 2000.	The authors reviewed 22 records and discuss two main topics: ‘narrative therapy and social justice’ and ‘women and ADHD’. The themes of the first topic were ‘narrative therapy and anti-oppressive practice’, ‘externalization as a social justice process’, and ‘externalization as highlighting personal agency’. The themes of the second topic were ‘psychiatric language and discourse’, ‘discursive gender oppression and ADHD’, ‘identity formation and ADHD’, and ‘diagnosis as an emergent event for change’.
Niemann, 2022 [21]	A Narrative Pastoral Counselling Approach to Giving a Voice to Children with ADHD with Anxiety	Dissertation, University of Pretoria, English	Qualitative literature investigation that aims to identify or develop a pastoral-narrative approach to ADHD children who suffer from anxiety (the principles of narrative therapy were applied to improve pastoral counseling for this population).	Narrative therapy (in the context of pastoral counseling) may help decrease the anxiety of ADHD children and lend meaning and support to their parents.
Nylund, 2000 [12]	Treating Huckleberry Finn: A new narrative approach to working with kids diagnosed ADD/ADHD.	Book, Jossey-Bass/Wiley, English	This book provides a comprehensive picture regarding the ideas and practical methods of the narrative approach in ADHD. The usage of the literary character of Huckleberry Finn (who possesses personality traits such as independency, nonconformity, and youthful innocence) echoes the social criticism embedded in Mark Twain’s book and illustrates how ADHD ‘symptoms’ can be re-framed as normative behaviors and even strengths.	Part 1 of the book outlines a critique of the ruling, bio-medical perception of ADHD, and part 2 elaborates on the solution offered by narrative therapy. This solution received the acronym SMART: Separating the problem from the child, Mapping the influence of ADHD, Attending to exceptions to the ADHD story, Reclaiming special abilities of diagnosed children, and Telling and celebrating the new story.
Nylund & Corsiglia, 1996 [19]	From deficits to special abilities: Working narratively with children labeled ‘ADHD.’	Chapter, Guilford Press, English	The book chapter discusses the theoretical ideas that underlie the narrative approach and presents in detail how narrative therapy is applied among children diagnosed with ADHD.	Key narrative therapy interventions for ADHD are presented alongside two clinical case examples that illustrate the basic ideas of the therapy (while contrasting them to the more traditional psychiatric approach).
Panahifar & Nouriani, 2021 [27]	The Effectiveness of Narrative Therapy on Behavioral Maladaptation and Psychological Health of Children with ADHD in Kerman	Journal Article, Iranian Journal of Pediatric Nursing, Persian	Quasi-experiment in which 30 children (aged 7–12) were allocated to narrative therapy (10 sessions) and control (no treatment).	Narrative therapy reduced behavioral maladaptation and increased the psychological health of children with ADHD.
Pedraza-Vargas et al., 2009 [65]	Narrative therapy in the co-construction of experience and family coping when facing an ADHD diagnosis	Journal Article, Universitas Psychologica, Spanish	Qualitative study among three families that have a child diagnosed with ADHD. The analysis of the families’ reports was conducted through the prism of narrative therapy.	The diagnosis of ADHD seemed to have led the families to construct a (problematic) dominant narrative that focused on the child’s symptoms and attracted prejudices and negative beliefs as well as parental guilt.
Realmuto & Lindberg, 2001 [31]	Treating Huckleberry Finn: A new narrative approach to working with kids diagnosed ADD/ADHD.	Book Review, Journal of the American Academy of Child & Adolescent Psychiatry, English	Review of the book “Treating Huckleberry Finn: A New Narrative Approach Working with Kids Diagnosed ADD/ADHD” by David Nylund.	This book review presents a relatively balanced position, with a slight tendency toward negativity. The authors appreciate the narrative approach and recommend considering some of its components in psychiatric care. However, they strongly oppose Nylund’s critical viewpoint of psychiatry and warn of its potential harm.
Rowlands, 2017 [24]	Through all them four letters, changes everything’: an exploration of the lived experience of children, with a diagnosis of ADHD, and their parents	Dissertation, University of Birmingham, English	Qualitative interviews with four children diagnosed with ADHD and one of their parents. In the spirit of narrative therapy, the interviews sought to separate the person from the problem (i.e., ADHD) and explore its effects on the interviewees.	The interviews with the children yielded two superordinate themes: ‘stories of suspicion, silence, and exclusion’ and ‘the way ADHD was perceived, experienced, and managed’ (corresponding subordinate themes can be found in Table 6.1 of the doctoral thesis). The interviews with the children yielded two superordinate themes: ‘stories of suspicion, silence, and exclusion’ and ‘the way ADHD was perceived, experienced, and managed’ (corresponding subordinate themes can be found in Table 6.1 of the doctoral thesis). The interviews with the parents yielded three superordinate themes: ‘a journey of pleading, proving, and compliance’, ‘stories of acceptance and validation’, and ‘ADHD is hard to live with’ (corresponding subordinate themes can be found in Table 7.1 of the doctoral thesis).
St James O’Connor et al., 1997 [66]	On the right track: Client experience of narrative therapy.	Journal Article, Contemporary Family Therapy: An International Journal, English	Qualitative research in which eight families that received narrative therapy were interviewed. The children (aged 6–13) of these families experienced a range of problems, including, for example, violence, school problems, grief, and ADHD.	The interviews yielded six major themes, including, for example, externalization, developing an alternate story, and personal agency. The therapy, according to the families, was ‘very effective’.
Thomas, 2004 [25]	Existential/experiential approaches to child and family psychotherapy (Chapter 4 in Comprehensive Handbook of Psychotherapy)	Chapter, John Wiley & Sons Inc., English	This book chapter reviews existential-experiential approaches to child and family psychotherapy. Three major approaches are discussed: ‘symbolic-experiential family therapy’, ‘emotionally focused family therapy’, and ‘narrative therapy’. The application of these approaches is discussed within a range of conditions, including, for example, sexual abuse, depression, parental divorce, and ADHD.	Basic principles of narrative therapy are presented alongside five leading methods in the treatment of children: externalization, playfulness, and creativity working with parents, collaborative coauthoring stories with clients, developing a counterplot to the problem-saturated story, and writing letters.

## 5. Conclusions

Without underestimating the difficulties and distress experienced by children diagnosed with ADHD, the potentially positive qualities associated with the ADHD-like personality (Section 4.3) may serve narrative therapists in constructing a more empowering and hopeful ‘health-side story’. This alternative story is not centered on deficits but rather on strengths, values, and healthy development (Introduction). Within this reframing, which also acknowledges the social pressures placed on children and parents, narrative therapists avoid formal medical language and potentially discouraging messages regarding the chronic and biogenetic nature of ADHD. Instead, they emphasize the existence of multiple storylines within every individual, thereby offering relief from parental guilt, reducing the risk of negative self-fulfilling prophecies, and facilitating pathways for growth and change (Section 3).

While narrative therapy still requires further empirical research to establish its effectiveness in the context of ADHD, the preliminary evidence presented in this scoping review, alongside the key interventions and conceptual insights, highlights its substantial potential as a compassionate and culturally sensitive psychotherapy, which departs from conventional paradigms, and invites a rethinking of how we understand and support children diagnosed with ADHD.

Importantly, the unique perspective on ADHD offered by narrative therapy may be justified—not only because of its empowering and non-pathologizing stance, but also in light of the absence of clear neurobiological markers for the diagnosis [67], and the ongoing debate and conflicting interests surrounding its scientific validity [32,68,69,70].

## Data Availability

The reviewed articles, dissertations, chapters, and books were collected from PsycNET, PubMed, Web of Science, ProQuest, and Google Scholar. Detailed information about the records included in this review is available in Table 1.
